# Implantation des sites de soins communautaires en République Démocratique du Congo: consécration d'un double standard dans l'accès aux soins

**DOI:** 10.11604/pamj.2013.14.158.2003

**Published:** 2013-04-24

**Authors:** Gisèle Mawazo Binti Dunia

**Affiliations:** 1 Agence Européenne pour le Développement et la Santé, Projet UNICEF, Haïti

**Keywords:** RDC, Site de soins communautaires, double standard, accès aux soins, Congo Democratic Republic, Site of community Care, double standard, access to care

## Abstract

Depuis 2005, la République Démocratique du Congo a amorcé l'implantation des sites de soins communautaires. Cette stratégie a pour objectif de rapprocher les services de santé des populations éloignées. Bien que cela parte d'une bonne intention, elle résulte, à notre sens, en une consécration d'un système de santé à deux vitesses. En effet, les populations vivant en ville ont accès à des soins prestés par des agents de santé formés alors que celles vivant en milieu rural reculé ont pour prestataires de soins des relais communautaires. Cette situation marginalise encore plus des populations dont la situation géographique est déjà préoccupante. Pourtant, la population est prête à parcourir des kilomètres en échange d'un service qu'elle estime de qualité.

## Introduction

Située au cœur de l'Afrique, la République Démocratique du Congo (RDC) est avec ses 2.345.000 km^2^ le troisième plus grand pays du continent. Sa population a été estimée en 2011 à environ 70 millions d'habitants dont près des trois quarts vit en milieu rural. Son système de santé repose sur les Zones de Santé (ZS) encadrée par le niveau intermédiaire sous le pilotage des services centraux du Ministère de la Santé Publique. La ZS est dirigé par une équipe cadre de la Zone de Santé (ECZ) ayant pour mission d'assurer la coordination et la gestion du système de santé local, notamment par l'amélioration de la couverture sanitaire et de la qualité des soins, la rationalisation du fonctionnement des Centres de Santés et de l'Hôpital Général de Référence (l'HGR est l'Hôpital de District), et l'organisation de la participation communautaire [1]. L'HGR offre un Paquet Complémentaire d'activités (PCA) en appui aux CS qui offrent chacun un Paquet Minimum d'Activités (PMA) à une population d'environ 10 000 habitants répartie sur une aire géographique de 5 à 8 km^2^ appelée Aire de Santé (AS). Les communautés locales ne se contentent pas d'utiliser les services offerts, elles apportent des ressources au CS et participent à leur gestion par le biais d'un comité de développement de l'aire de santé (CODESA).

Seulement 20% de la population utilise couramment les services de santé [2]. Aussi, les taux de mortalité infantile et infanto juvénile de la RDC sont parmi les plus élevés d'Afrique Subsaharienne. Ils sont respectivement de 92 et de 148 décès pour 1000 naissances vivantes [3]. La sous-utilisation des formations sanitaires s'explique en partie par une faible accessibilité géographique des populations aux soins [4]. Pour l'améliorer, des sites de soins communautaires - points de prestation des soins fournis par deux relais communautaires (RC) formés et supervisés - ont été implantés dans les zones dites d'accès difficile mais, la qualité des soins qui y sont dispensés justifie-t-elle leur installation’ Les principes de bioéthique fournissent un cadre général qui peut être appliqué à l'analyse de ce dilemme. Cette analyse doit refléter à la fois les normes internationalement reconnues et les valeurs culturelles locales pertinentes [5].

## Cadre théorique

Les populations vivant dans les milieux enclavés ou éloignés des Formations Sanitaires (FoSa) doivent se contenter des services des Relais Communautaires alors que les populations vivant en milieu urbanisé, proches des FoSa (moins de 5km ou d'une heure de marche), ont l'opportunité de consulter des formations sanitaires tenues par des professionnels de la santé.

Par définition, le RC est un homme ou une femme volontaire, habitant le village ou la rue et choisi par les habitants de cette entité, qui assure le pont entre les individus, leurs familles et le service de santé. C'est un volontaire non rémunéré à qui est confiée la gestion des problèmes de santé de l'enfant considérés comme mineurs, mais aussi d'autres membres de la communauté. Il pourrait être considéré comme personnel de santé qui d'après l'Organisation Mondiale de la Santé (OMS) est l'ensemble des personnes exerçant des activités dont l'objet essentiel est d'améliorer la santé, y compris les différents aidants familiaux, le couple patient-soignant, le personnel à temps partiel, les bénévoles et les agents communautaires. Plus particulièrement, l'on désigne par le terme “agent ou travailleur de santé” tout personnel de santé rémunéré pour sa prestation de soins [6]. Le RC à qui est confié la gestion du site de soins communautaires n'est officiellement pas rémunérée. Il ne répond pas à la deuxième partie de la définition, et n'est donc pas un agent de santé, la seule compétence exigée lors de sa sélection étant de savoir lire et écrire.

Pour analyser l'utilisation du personnel non qualifié en RDC par le Ministère de la Santé, la notion de “*double standard des soins*” [7] peut être évoquée. Empruntée à la recherche sur les sujets humains, cette notion stipule que “...les patients du groupe témoin devraient recevoir un niveau universel de soins pour la maladie étudiée. Lorsqu'il est inopportun d'offrir un tel niveau de soins, il faudrait au minimum offrir la meilleure intervention disponible habituellement dans le contexte du système national de santé publique...” [8]. Ce concept a été consacré par les modifications apportées dans la version 2000 de la déclaration d'Helsinki qui est un ensemble de principes d'éthique régulièrement mis à jour, dont l'objectif est de fournir des recommandations aux médecins et participants à la recherche sur l'être humain. Il a suscité des nombreuses réactions des défenseurs de la bioéthique car dans un pays à revenu faible, le standard local de soins est parfois une absence de soins pour les participants formant le groupe témoin dans une étude d'intervention (essai clinique). La demande des bénéficiaires est alors influencée par leurs perceptions des problèmes de santé et des solutions qui peuvent y être apportées. Ces solutions qui déterminent l'itinéraire thérapeutique vont des soins auto-administrés à la consultation des spécialistes.

## Soins auto administrés et soins délivrés par les agents communautaires

### Soins auto administrés

Le “self care” ou soins auto administrés a vu le jour dans les années 70 avec l'avènement de la promotion de la santé. Il s'agit d'un processus au cours duquel un individu ou un aidant proche mène des actions de promotion de la santé: hygiène et alimentation saines, pratique de l'activité physique, soins aux malades chroniques (diabète, hypertension artérielle,...). Dans une étude qualitative menée en Ecosse en 2004 [9], la majorité des personnes interrogées préfèrent, pour des problèmes de santé mineurs, des soins auto administrés aux consultations de médecins. D'autres ont une certaine propension à partager les prescriptions ou les conseils sans se référer aux professionnels de santé. Ce comportement se retrouve principalement dans les cas suivants: (i) utilisation en famille du reste des médicaments d'une prescription antérieure, (ii) partage des médicaments avec un ami/proche lorsque les problèmes de santé sont jugés identiques, (iii) offre de médicaments avec la volonté “d'aider un ami”. Il s'agit d'un comportement à risque conduisant souvent à des comorbidités importantes [10].

### Participation communautaire dans les pays en développement

La participation communautaire en santé est prônée par la Déclaration d'Alma Ata [11], renforcée par plusieurs autres déclarations et documents [12–15]. Elle prend plusieurs formes selon les pays: contribution financière et/ou matérielle au fonctionnement des formations sanitaires, cogestion, visites à domiciles, détection et référence des problèmes de santé dans les familles,' Cette implication va évoluer au fil des années avec le développement de la Prise en Charge Intégrée des Maladies de l'Enfance (PCIME) dans les années 90. En 1997, la réunion de coordination de la PCIME tenue à Santo Domingo reconnait que l'amélioration de la qualité des services dans les FoSa est certes indispensable mais, ne pourrait à elle seule contribuer à réduire de façon significative la morbimortalité infantile. Elle recommande une approche communautaire de la PCIME (PCIME communautaire) [16]. C'est dans ce cadre qu'en Inde, un programme de la Society for Education, Action and Research in Community Health [17] dispense des soins de santé de base aux bébés dont les mères accouchent à domicile depuis quelques années. Une formation ainsi que du matériel médical de base est remis aux agents de santé communautaires à cet effet. D'un bon rapport qualité/prix, le modèle a inspiré des expériences à base communautaire visant à sauver la vie des nouveaux nés dans des communautés pauvres de plusieurs pays africains. La prise en charge s'est étendue, peu à peu, des nouveaux nés aux enfants de moins de 5 ans présentant des problèmes de santé dits mineurs. Dans la majorité des cas, l'encadrement d'un personnel compétent et motivé est nécessaire pour répondre aux besoins en matière de santé [4, 12, 18].

## L'introduction des sites de soins communautaires en RDC

Soutenu par ses partenaires au développement, le Ministère de la Santé, a amorcé l'intégration de l'approche “sites de soins communautaires” en vue de rapprocher les services de soins de la population en 2005. Les objectifs poursuivis sont [4]: (i) Assurer les premiers soins adéquats aux populations n'ayant pas un accès géographique aux structures de soins; (ii) Assurer la référence des cas avec signes de danger/alerte; (iii) Améliorer la disponibilité et l'utilisation rationnelle des médicaments essentiels génériques (MEG) de qualité; (iv) Améliorer la notification des cas de maladies ainsi que la surveillance à base communautaire; (v) Améliorer les pratiques clés en rapport avec la survie de l'enfant. Cinquante-deux ZS aussi bien rurales qu'urbaines - y compris 6 ZS de la ville de Kinshasa - sur les 515 que compte le pays - ont été progressivement incluses dans cette approche. Depuis lors, 716 sites de soins ont été mis en place [19].

Pour organiser la prise en charge des patients dans les sites, les RC disposent de médicaments, outils de gestion et autres intrants. Si les soins dispensés dans ces sites sont en principe gratuits, les patients paient les médicaments qui sont fournis par les CS. Leurs prix sont fixés avec une marge bénéficiaire, en accord avec l'Infirmier Titulaire (IT) et l'ECZ, en veillant à ce que cette dernière ne rende pas les médicaments inaccessibles financièrement. Le comité de gestion du site (COGESITE) s'assure périodiquement du recouvrement des coûts et de la gestion des intrants et l'ECZ, un suivi régulier, des supervisions et l'approvisionnement en intrants. Pour chaque patient pris en charge, le RC doit organiser des visites à domicile régulières (au minimum 3 par patient) afin de s'assurer du suivi des recommandations, notamment la compliance au traitement [4].

Le suivi post formation des RC est effectué par des formateurs du niveau central accompagnés si nécessaire des équipes provinciales et ZS [20]. La supervision est organisée une fois par mois par l'IT du CS dans chacun des sites de soins communautaires de son AS. Elle se fait par revue documentaire et observation. Si le temps est limité, le superviseur peut emporter les fiches qu'il examinera dans son bureau. L'accompagnement par le superviseur ou l'animateur communautaire de la ZS n'est indispensable que lorsque des manquements sont constatés et notifiés par l'IT.

Une évaluation de la stratégie a été dirigée en 2010 par l'organisation Maternal and Child Health Integrated Program financé par l'USAID [19]. Elle montre des succès: engagement politique, renforcement des liens avec la communauté, disponibilité des services de qualité; mais aussi des goulots d'étranglement: faible implication des cadres provinciaux et des ZS, ruptures de stock, revendication d'une motivation financière des RC, dépendance à l'aide financière étrangère. Depuis leur mise en place, les sites ont pris en charge des patients pour lesquels il a été suspecté, selon les définitions de cas: un paludisme, une diarrhée simple, une pneumonie, une toux ou une malnutrition légère. La plupart des fiches de prise en charge (80%) montrent une concordance entre les signes et la classification faite par le RC. 7% des cas consultés ont été considérés comme nécessitant une prise en charge au niveau des centres de santé, parmi lesquels 85% ont été effectivement référés [21].

## Appréciation des résultats intermédiaires

### L'inefficacité de la stratégie adoptée

En présentant comme succès la disponibilité des services de qualité alors qu'elle note des ruptures de stocks, l'évaluation intermédiaire est contradictoire. Une interprétation des chiffres ci-dessus pourrait être que les 4,5% de personnes (85% de 7%) reçues au CS constituent la vraie population pour laquelle les sites de soins communautaires ont évité la survenue de problèmes graves. En effet, la plupart des problèmes de santé se résolvent spontanément ou sont pris en charge en famille [22]. Seulement une faible proportion requiert l'intervention d'un professionnel de santé, les problèmes pris en charge par le système de santé ne constituant que le sommet de l'iceberg. A contrario, on pourrait se demander ce que sont devenues les 20% de personnes chez lesquelles un diagnostic erroné a été posé. Y a-t-il eu des cas de guérisons spontanées ou plutôt des décès’ Combien de complications à moyen ou long terme surviendront-elles chez ces personnes’ Certains patients consultaient certainement pour des épisodes aigues de pathologies chroniques, mais il est difficile de savoir s'ils sont revenus dans le système par une autre porte d'entrée. On sait toutefois que les populations non couvertes par des CS tendent à recourir aux services des officines tenues par des non professionnels, des vendeurs de médicaments de rue, charlatans et autres escrocs. Le site de soins communautaire peut alors être considéré comme un moindre mal. Il est vrai que l'idée initiale du volet communautaire de la PCIME qui était de sensibiliser la population à l'utilisation des services de santé, aux pratiques clés de l'hygiène et de l'alimentation, était intéressante. L'initiative a évoluée vers l'encouragement de la prise en charge des problèmes de santé par des membres de la communauté, négligeant dès lors les risques iatrogènes et la probabilité importante que des soins prestés par des non professionnels soient inefficaces. Pourtant, ces risques ne peuvent être minimisés par les supervisions des IT qui ne bénéficient toujours pas eux-mêmes de l'encadrement des superviseurs de ZS dans un processus d'amélioration continu de la qualité. Ainsi, l'administration des antibiotiques et antipaludiques par des mains non expertes pourrait entraîner des effets secondaires majeurs difficiles à gérer par le RC, sachant que les structures de soins sont par définition éloignées des sites de soins communautaires.

Par ailleurs, l′utilisation des services de santé dépend de différents facteurs, dont la perception par les clients potentiels de la maladie (causes, symptômes, conséquences, vulnérabilité), de la qualité des soins offerts et l′accessibilité des services. Si l'installation des sites de soins communautaires peut contribuer à améliorer l'accessibilité géographique, elle n'a aucune influence sur l'accueil, l'accessibilité financière et culturelle, la qualité technique des soins. Des études ont montré que l'utilisation des accoucheuses traditionnelles dans les villages éloignés des maternités n'a pas produit les résultats escomptés. En l'absence d'amélioration des services de santé, la mortalité maternelle n'a pas diminué comme attendu [23]. Une méta-analyse effectuée en 2007 a également montré que l'utilisation des RC n'est pas la solution pour rapprocher les services de la population dans les pays à système de santé faible [24]. L'une des grandes leçons tirées de la mise en place des programmes spécialisés est la difficulté d'améliorer à long terme les pratiques sanitaires sans services de base assez performants pour soutenir et consolider les interventions.

### L'inéquité dans l'accès aux soins

On peut comprendre le souci, par équité horizontale, de rapprocher les services de santé de la population. Mais la création de “semi infirmiers” ou pire “semi aides-soignants” pour des populations que la situation socioéconomique marginalise déjà ne se justifie pas en RDC. Même s'il est difficile de dire combien de médecins et d'infirmiers prestent en RDC aujourd'hui, la qualification et le niveau technique des relais communautaires sont insuffisants pour constituer une alternative. Près de 1.500 médecins sortent chaque année des seules Universités de Kinshasa et de Lubumbashi. Les Instituts des Techniques Médicales, environ 362 dans le pays, forment chaque année près de 7.000 infirmiers [1]. Aussi, les ressources consacrées à la création des sites de soins communautaires peuvent être utilisées pour la construction/réhabilitation de structures de santé respectant des plans rationnels d'extension de couverture, car “les effets pervers de cette inflation du personnel commencent déjà à se faire sentir sur terrain. On assiste depuis quelques années à une multiplication du nombre des structures de santé pour la plupart créées par le personnel de santé formé en surplus et qui n'a pas trouvé du travail dans une structure formelle”. En limitant cette multiplication anarchique relevée dans son propre document de Stratégie de Renforcement du Système de Santé, le Ministère de la Santé congolais veillerait à une offre de service de qualité aux populations. Garant de la santé de toute la population, il ne devrait pas donner son aval pour un accès aux soins à deux vitesses, l'état ayant le devoir de préserver l'équité. La qualité de l'offre de soins ne devrait pas dépendre, dans un même pays, province, voire ZS, du milieu de résidence (urbain ou rural) ou de la distance par rapport à une structure sanitaire aux normes. On peut d'ailleurs se demander si l'installation des sites de soins communautaire n'a pas été fortement influencée par une tendance de certains acteurs à promouvoir les concepts à la mode en Santé Publique. Influencés par l'agenda international, ils se concentrent essentiellement sur la réduction de la mortalité et de la morbidité et ‘œuvrent pour la multiplication des campagnes de masse et l'offre de soins préventifs et curatifs gratuits. Bien que le fait de cibler les populations les plus pauvres et vulnérables soit louable, il ne faudrait pas donner l'impression que les FoSa offrant en routine des services de qualité centrés sur l'individu, ne sont réservées qu'aux populations urbaines et à une élite qui peut se les payer.

## Les perspectives à redouter

La formation des volontaires de la Croix Rouge (secouristes) dans les années 90 a entrainé en RD Congo la création de nouvelles structures de soins dirigées par ces derniers. Cette dérive pourrait se répéter si dans leur sillage, certains relais communautaires formés se considèrent désormais comme des infirmiers à part entière. Sous la couverture d'un quitus du Ministère de la Santé, ils prendraient en charge des patients au-delà de la cible prescrite et retiendraient des patients même lorsque des problèmes de santé classés mineurs au départ s'aggravaient avec le temps. Par ailleurs, une utilisation abusive par les RC des antibiotiques et antipaludiques qu'ils détiennent pourrait être à l'origine de l'émergence de résistances. La marge de manœuvre des agents de santé étant réduite notamment en termes de coût et disponibilité des molécules de seconde ligne, la prise en charge des patients transférés au CS ou à l'hôpital sera plus coûteuse, l'accès aux soins indispensables davantage réduit.

En admettant que certains RC des sites de soins peuvent devenir réellement compétents par seul apprentissage, ils seraient malgré tout limités par la difficulté à trouver des solutions en cas de problèmes non encore rencontrés. Pourtant, certains auront tendance à outrepasser leurs limites par ignorance des dangers encourus ou par goût du profit. Ils devraient donc bénéficier d'un encadrement de proximité d′une personne plus qualifiée. Cet encadrement est confié aux ECZ qui exécutent déjà difficilement les plans de supervisions et suivi qu'elles élaborent habituellement. Aussi, le nouveau besoin d'encadrement des sites ne peut être couvert que si un bailleur de fond en assure le financement, avec comme risque la négligence de certaines autres activités de routine. L'administration des soins par les RC pourrait souffrir de nombreuses autres entorses [4, 22–24]: Compétition entre les FoSa et ces bénévoles qui acceptent de consacrer une partie de leur temps aux activités d'intérêts communautaires: certaines occupations habituelles du RC souffrent du fait qu'il consacre beaucoup de temps à la prestation de soins lorsqu'il est désigné responsable de site. Il peut être tenté de s'auto rémunérer à travers les consultations ou la vente illicite des médicaments; création de structures parallèles aux CODESA: l'implantation des sites de soins communautaires a été accompagnée de la création des COGESITE autonomes qui ne participent pas à la cogestion des Centres de Santé. Ils pourraient contribuer à fragmenter ou diluer l'information à donner à la communauté dans le cadre du marketing social.

## Conclusion et recommandations

La mise en place des sites de soins communautaires pour réduire l'inaccessibilité géographique comporte d'énormes risques, le nombre d'agents qualifiés et motivés pour assurer un suivi régulier et un encadrement des non-professionnels commis à l'offre des soins étant limité. Comme par ailleurs le nombre de médecins et infirmiers est “en forte hausse depuis le début des années 2000 avec une inflation des écoles d′enseignement médical” [1], il serait judicieux d'arrêter la mise en place de sites de soins communautaires. En attendant une évaluation rigoureuse de leur impact, seuls des sites bénéficiant déjà d'un encadrement rigoureux des ECZ pourraient continuer à fonctionner. Des déplacements périodiques, stratégies avancées ou cliniques mobiles devraient être organisés par le personnel qualifié pour les populations non couvertes. Le foyer étant le premier lieu de prise en charge des maladies infantiles, il faudra également, sans encourager l'usage d'antibiotiques, améliorer la maitrise par les mères et autres proches des premiers gestes thérapeutiques ainsi que la reconnaissance des signes de danger, pour éviter un recours tardif aux services de santé. Les programmes scolaires devraient aussi intégrer les bonnes pratiques éprouvées dans l'amélioration de la santé.
